# Cesarian Operation

**Published:** 1850-09

**Authors:** John Travis

**Affiliations:** Mansfield, Tennessee


*Cesarean Operation.* By JOHN TRAVIS, M. D., of Mansfield, Tennessee.

The Cesarian Operation is a rare one, and, when performed, it has generally proved unsuccessful, more so in Europe than in America.

In the year 1841, I was called to a lady aged 28 years, who was in labor with her third child, which could not be delivered in the ordinary way on account of malformation of the fœtus. I performed the operation in the usual manner, and the mother and child are both living.

The mode of operation is so well described by authors, and so generally understood by physicians, that a description here is deemed unnecessary.

The case is briefly reported merely to show that it was successful.

We have inserted the above communication, more for the sake of cautioning the younger members of the profession against the dangerous doctrine it inculcates, than for any merit it possesses in iteslf.

The author has stated that he performed what is confessedly one of the most dangerous operations in surgery, upon a woman having a well formed pelvis, (as witnessed by her two previous labors,) merely on account of an unnamed malformation of the fœtus, and one too that did not compromise its life.

We aver, and in this we are supported by the highest authorities, that no man has a right to perform this operation under any circumstances, unless it be clearly established that delivery per *vias naturales* is more dangerous to the mother than the abdominal section.

Cazeaux states "that the operation may be performed on the living female, whenever the natural passages through which the child has to pass are too narrow, or so obstructed, that a delivery by symphysiotomy, or the application of the forceps is, absolutely impossible; and when the mutilation of the child itself, would not permit its extraction without exposing the mother to the greatest dangers."

Dr. Meigs says. "I should be glad, in the most emphatic manner, to enter my protest upon the Records of Obstetrics, against the Cesarean operation being performed with any other view than those relative to the conservation of the mother, with the salvo always, that to save the child is great additional good fortune. I believe that he who performs the Cesarean section upon views relative chiefly to the conservation of the fœtus, flies in the face of the soundest doctrine; and I cannot understand how the conscience of such an operator should ever be appeased under the pungent reflections that must follow a death not rendered inevitable by the exigencies of the patient."

Dr. Ramsbotham, in speaking on this subject, declares that "in the British Isles, out of nearly thirty operations, we only have three instances of recovery on record." And further on: "The fact is not to be concealed, that in different parts of Europe, and especially in Catholic countries, has the operation many times been had recourse to, under circumstances in which no British practitioner would have considered himself warranted in proposing it; where indeed there has existed sufficient available space in the pelvis to admit of the extraction of the fœtus *per vias naturales.*" And in a note he says: "It would be unfair to adduce in confirmation those cases in the latter half of the sixteenth century, the second, third, fifth and sixth of Rousset, which came under his own observation, and the ninth of Caspar Bauhine all which women brought forth live children, *per vaginam* subsequently, even if they are correctly reported; because they belong to the age of *barbarous surgery.*" And still further on he says: "In Britian we never substitute it for craniotomy by choice, but only have recourse to it when no other mode of delivery is practicable." p. 263.

Velpeau says, p. 506—"It may be stated, therefore, that up to the present day, the Cesarean operation has proved fatal in at least one out of five cases, and that Tennon was mistaken in asserting that since the time of Bauhine, it has been performed at the Hotel Dieu on seventy women, who recovered. By the report of J. Burns and S. Cooper, it appears that not a single well attested case of its successful performance has occurred in Great Britian, although the number of operations amount to fifteen or twenty. I think these details will suffice to exhibit the operation to young practitioners in all its importance, and to prevent them from resorting to it in any case, save when the necessity is absolute."

Gooch declares that "the operation is performed only when the deformity of the pelvis is so great, that even after perforating the head and removing the bones of the cranium there is not sufficient space for the extraction of what remains of the child." p. 214.

These authorities, hastily collected, are in our opinion sufficient to establish the danger of the operation, and to dissuade any one from undertaking it, except under the circumstances pointed out. But putting out of the question, the danger of the mother, it should be remembered that the safety of the child is also compromised, for the most stubborn advocates of the Cesarean section are forced to acknowledge that they are not always fortunate enough to extract a living child, even when the operation is performed at the time of their own selection. And even should the child survive, can it fill the void which is almost certain to be created by the death of its parent?

If we have censured the author of the above report unjustly, we can only plead in justification, the meagre details with which we are furnished; but as it now stands, we are compelled, both on the ground of humanity and scientific surgery, to enter our protest against the operation as unwarrantable, and our caution lest others should "go and do likewise."—Ed. Examiner.]

